# Two *Leishmania* species separation targeting the ITS-rDNA and Cyt b genes by developing and evaluating HRM- qPCR

**DOI:** 10.1590/0037-8682-0186-2022

**Published:** 2022-12-16

**Authors:** Elnaz Alaeenovin, Parviz Parvizi, Seyedeh Maryam Ghafari

**Affiliations:** 1 Pasteur Institute of Iran, Molecular Systematics Laboratory, Parasitology Department, Tehran, Iran.

**Keywords:** Cyt b, ITS-rDNA, PCR, Real-time PCR, HRM

## Abstract

**Background:**

Incidence of Cutaneous Leishmaniasis as an infectious and neglected disease is increasing, for the diagnosis of which several traditional methods and conventional PCR techniques have been developed, employing different genes for species identification.

**Methods:**

*Leishmania* parasites were sampled, DNA was extracted, and new specific and sensitive primers were designed. Two ITS-rDNA and Cyt b genes were targeted by qPCR using the High- Resolution Melting method to identify *Leishmania* parasites. The standard curves were drawn, compared, and identified by high-resolution melting curve analysis.

**Results:**

Melting temperature and Cycle of Threshold of ITS-rDNA was higher than Cyt b but Cyt b was more sensitive than ITS-rDNA when *Leishmania major* and *Leishmania tropica* were analyzed and evaluated. By aligning melt curves, normalizing fluorescence curves, and difference plotting melt curves, each *Leishmania* species was distinguished easily. *L. major* and *L. tropica* were separated at 83.6 °C and 84.7 °C, respectively, with less than 0.9 °C of temperature difference. Developing sensitivity and specificity of real-time PCR based on EvaGreen could detect DNA concentration to less than one pmol.

**Conclusions:**

Precise identification of *Leishmania* parasites is crucial for strategies of disease control. Real-time PCR using EvaGreen provides rapid, highly sensitive, and specific detection of parasite’s DNA. The modified High-Resolution Melting could determine unique curves and was able to detect single nucleotide polymorphisms according to small differences in the nucleotide content of *Leishmania* parasites.

## INTRODUCTION

Leishmaniasis, one of the neglected tropical and subtropical diseases, has been considered based on the medical importance; besides, visceral leishmaniasis is potentially lethal^1^ ^-^ ^3^. From all known *Leishmania* species, some are medically important. In Cutaneous Leishmaniasis (CL) the lesions caused by different *Leishmania* species, which are usually large but painless unless a secondary bacterial or fungal infection occurs. The skin manifestations of the disease in final stages can usually lead to obstruction or degradation of the pharynx, larynx, and nose^4^. 

CL includes ZCL (Zoonotic Cutaneous Leishmaniasis) and ACL (Anthroponotic Cutaneous Leishmaniasis). These important tropical diseases have many impressive effects on the human health. The mammalian host(s) and human were infected by different *Leishmania* parasites in the Old World. 

The vertebrate animals were bitten by female sand flies and thus developed ZCL. Humans were involved in *Leishmania* parasites and they were involved ACL by humans oppositely^5^ ^,^ ^6^.

In general, precise identification of infectious agents of leishmaniasis and their clinical manifestations are needed in reservoir hosts, vectors, and humans. Well known and practical methods for the detection and recognition of *Leishmania* species were employed. The Giemsa staining, clinical indications, vector estimations, in vivo pathogenicity, growth in media, and isoenzyme were used for distinguishing between different *Leishmania* parasites. Later PCR, RFLP (Restriction Fragment Length Polymorphism), sequencing, and phylogenetic analysis were applied^1^ ^,^ ^7^.

High- Resolution Melting (HRM) is an efficient and cost-effective procedure that eliminates the risk of laboratory contaminations. Real-time PCR using HRM can firmly identify most well-known *Leishmania* species by employing housekeeping nuclear ITS-rDNA and mitochondrial Cyt b genes^3^ ^,^ ^8^ ^-^ ^10^. This method is inexpensive, rapid, and has high-performance detection; it requires no electrophoresis analysis, reducing risk of contamination as well as significantly decreasing workload, time, and cost. HRM is a relatively new analysis technique that allows direct identification of PCR amplicons. In addition, advantage of qPCR over conventional PCR is that initial DNA concentration is determined with high precision and sensitivity. The results using HRM-qPCR can be qualitative and/or quantitative^11^ ^,^ ^12^.

ITS-rDNA is a housekeeping gene and Cyt b is an enzymatic and expression gene. 

For precise identification of *Leishmania* parasites, two housekeeping ITS-rDNA and mitochondrial Cyt b genes were targeted and HRM-qPCR assay was modified and developed. This was an important purpose of this research. Accuracy, speed, and especially affordability as well as low cost, were the secondary purposes of this study. Recently, it has been shown that the colors of EvaGreen and SYTO rather than SYBR Green are more specific and more susceptible for measuring DNA quantification in qPCR; therefore, this color was used in this investigation^13^ ^,^ ^14^.

## METHODS

### Sampling of *Leishmania* isolates and DNA extraction

**SUPPLEMENTARY FIGURE 1:**
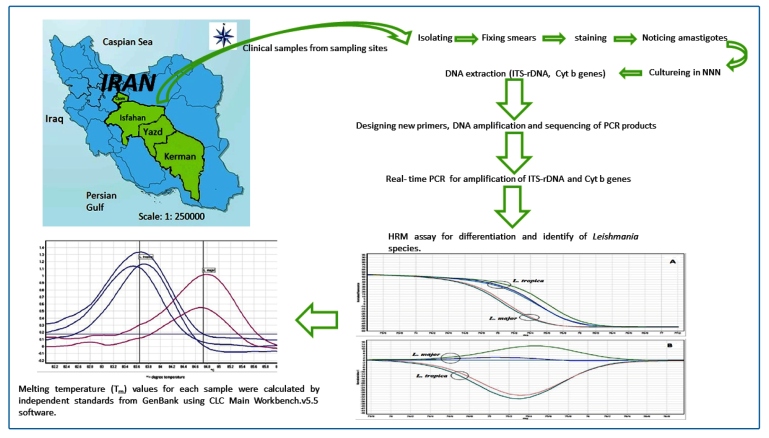
Graphical abstract highlighting important methodical aspects of this research.

Different *Leishmania* parasites from different locations of Iran were employed including four strains that were represented in more than 160 collected samples. Following extraction and sequencing, four haplotypes were found and three standard strains of *Leishmania* from other places (WHO1 (MHOM/SU/73/5ASKH), K27.11 (MHOM/SU/74/K27) from Soviet Union and FR05 (MHOM/IL/80/fredlin) from Italy) were sampled with the DNA extracted for precise recognition of the *Leishmania* parasites using the ITS-rDNA and Cyt b genes (Supplementary Figure 1 **)**, (Table 1). 

**TABLE 1:** *Leishmania* species identification using PCR applying ITS-rDNA and Cyt b genes and separation by HRM-qPCR.

*Leishmania species*	UCSA *	Origin	HRM- qPCR			
	(International code)		ITS2		Cyt b	
			Tm	CT	Tm	CT
*L. major*	AB03	Abarkouh/Iran	83.48	23.86	65.62	33.35
	WHO1**	Soviet Union	84.95	23.85	65.72	27.24
	(MHOM/SU/73/5ASKH)					
	FR05	Italy	84.83	23.88	65.33	17.74
	(MHOM/IL/80/fredlin)					
*L. tropica*	KE06	Kerman/Iran	83.92	23.92	65.27	33.5
	K27.11	Soviet Union	83.83	23.93	66.33	21.29
	(MHOM/SU/74/K27**)					
	KE12/HU12	Kerman/Iran	83.92	23.98	65.40	29.71
	KE07	Kerman/Iran	83.93	23.96	65.45	-

**CT:** Cycle of Threshold; **Tm:** melting temperature. *unique code of sample analysis (UCSA); **standard WHO reference strain.

Clinical specimens were isolated and smeared according to the Declaration of Helsinki, Ethical Principles for Medical Research Involving Human Subjects protocol, and Ethical Committee Guidelines of Pasteur Institute of Iran^15^ ^,^ ^16^. The fixation of smears was followed by Giemsa staining, so that amastigotes were seen using light microscope (×1000). 

Culturing samples in Novy-MacNeal-Nicolle (NNN) medium were carried out, then incubation was done and samples were checked for the observation of the presence and growth of promastigotes. The isolation of promastigotes was followed by transferring to RPMI-1640 media and the counting of the parasites in a Neubauer chamber (about 10 million promastigotes per/ml).

DNA was extracted using a tissue extraction kit (Takapozist, Iran). The promastigotes were washed using phosphate buffer after one week of culturing. Proteinase K (20 μl) was added to the parasites (200 μl). Samples were subjected to 56 ºC hot bath. Absolute ethanol was added. Columns were washed with releasing buffer to obtain purified extracted DNA.

### Designing new primers and DNA amplification of *Leishmania* parasites

Cyt b is mitochondrial; it is an enzymatic or expression gene. It has a 20-50 copy number. The infection was detected using forward primer (LCBF1F) (5' GGTGTAGGTTTTAGTTTAGG 3') and reverse primer (RLCBR2)(5'CTACAATAAACAAATCATAATATACAATT 3')^17^. After cloning the fragment, two species were differentiated with new designed primers using the CLC DNA workbench 5.5 software (CLC, Bio-QIAGEN, Aarhus, Denmark). For the newly designed primers, following criteria were considered: 1- the primer length has to be between 11-30 bp; 2- the hybridization temperature of the primers has to be between 58-60 °C; 3- the candidate region has to be specific for *Leishmania* genus; 4- the prediction of the temperature regarding the mutations has to be intended for detecting region. Then newly designed primers were used in HRM-qPCR: forward primer (5' ACTCACGGCCTCTAGGAATGA3') and reverse primer (5' AGAATTTCACCTCTGACGCCCCAGT3'). 

ITS-rDNA is a chromosomal, housekeeping gene. It has 40-200 copy numbers. Using forward primer (ITS1F) (5’ GCAGCTGGATCATTTTCC 3’) and reverse primer ITS2R4 (5’ ATATGCAGAAGAGAGAGGAGGC 3’), 480 bp fragment was amplified and the infection was detected^18^. Then another set of primers was used in HRM-qPCR: (ITSmF1) (5’ GTGTGGAAGCCAAGAGGAGG 3’) and reverse primer (ITSmR2) (5’ GCAAGCACCAGAGAGGAGT 3’); the size of the fragment (microsatellites of ITS-rDNA gene) was 160 bp, including primers^19^. 

**FIGURE 1:**
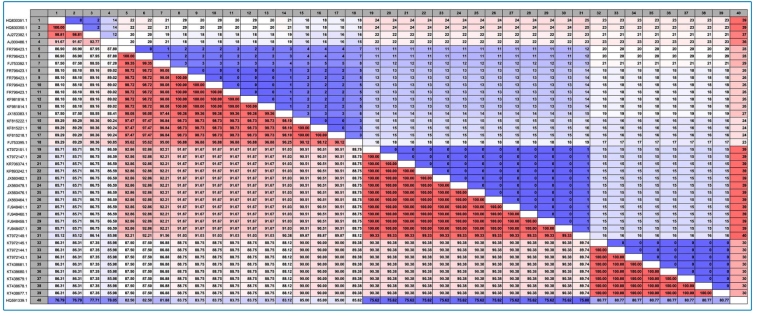
Comparison among cover percentage of studied species in this research and other species from GenBank using the CLC Main Workbench 5.5 software.

For these newly- designed primers, the similarity, sensitivity, specificity, proximity, and coverage percentages were evaluated with *L. major*, *L. Infantum*, *L. turanica, L. tropica*, *L. donovani,* and *L. gerbili* (Figure 1). For each PCR reaction, a total volume of 25 μl was utilized containing 0.4 pmol of each primer, 1X PCR buffer (750 mM of Tris-HCl, pH: 8.8 at 25 °C, 200 mM of (NH4)2SO4 and 0.1% Tween-20), 2.5 mM of MgCl2, 0.5 mM of deoxy ribonucleotide triphosphate (dNTP), 1 U of Taq polymerase (CinnaClon Co., Iran), and 10 ng/reaction of each DNA. For the newly designed primers, PCR program was as follows: initial denaturation at 94 °C for 4 min, followed by 35 cycles at 94 °C for 30 s, annealing for each gene (Cyt b: at 50 °C for 60 s; ITS-rDNA: at 58 °C for 45 s), and extension at 72 °C for 1 min, as well as final extension at 72 °C for 5 min. After purification, PCR products were sent for sequencing by an ABI PRISM™ 310 automated sequencer (Applied Biosystems, USA), using reverse and forward primers. Sequences were edited and aligned via Sequencher 3.1.1 and the CLC Main Workbench 5.5 software (CLC, Bio-QIAGEN, Aarhus, Denmark).

### ITS-rDNA and Cyt b genes amplification by Real- time PCR

Cyt b was amplified applying newly designed primers using Real-time PCR. ITS-rDNA was amplified according to the protocols of Parvizi and Ready, 2008. The mapping genes of these primers and time-temperature schedule were determined using the CLC DNA workbench 5.5 software (CLC, Bio-QIAGEN, Aarhus, Denmark). The protocols were modified and performed to detect *Leishmania* species employing the HRM method by Rotor-Gene 6000 Series Software 1.7. The modified protocol of HRM was followed: first, holding 95 °C for 15 min by using 4 µl of 5x HOT FIREPol® EvaGreen® qPCR Mix Plus (Solis Bio Dyne, Estonia). In the first stage, DNA denaturation of template and activation of polymerase enzyme were carried out at 95 °C for 15 s. In the second stage, DNA replication was performed in 45 cycles, at 55 °C for 30 s and at 72 °C for 30 s, with final extension cycle at 72 °C for 300 s. The separation curve or melting curve were generated at 95 °C for 60 s or 60 °C for 60 s. Finally, to obtain HRM from 65 to 95 °C, with every 0.1 °C rise, two seconds were paused and finally all of these steps were optimized. GC content, length, sequence, and heterozygosity of DNA affect the melting characteristics of a PCR product. PCR products were controlled by saturated EvaGreen color and were fluoresced in the presence of two-strand of DNA.

The standard samples for qPCR and re-sequencing of ambiguous sites were prepared according to Ghafari et al, 2020 with some modifications^20^.

### Absolute quantification qPCR and improving HRM

*Leishmania* parasites were identified using newly designed primers by high-resolution melting-curve analysis. Amplification of *Leishmania* DNA for real-time PCR was set for a total volume of 20 μl containing 0.7 µl of each designed forward/reveres primers, EvaGreen 5x (Yekta tajhiz azma), 12.6 µl sterile DNase/RNase-free water, and 10 ng/reaction of each DNA sample. Functionality and sensitivity of HRM assay were developed to distinguish and recognize the *Leishmania* species. The results of HRM dissociation were compared with the standard PCR data. Melting temperature (T_m_) values for each sample were calculated by independent standards from GenBank using the CLC Main Workbench.v5.5 software (CLC, Bio-QIAGEN, Aarhus, Denmark).

## RESULTS

### *Leishmania* identification by employing ITS-rDNA and Cyt b genes

Two *Leishmania* species from Iranian sources and some *Leishmania* reference strains were precisely identified by employing ITS-rDNA and Cyt b genes (Table 1). 

The sensitivity and specificity of each gene were identified and evaluated to accurately identify *Leishmania* species (*L. major* and *L. tropica)*. 

T_m_ and Cycle of Threshold (CT) of ITS-rDNA and Cyt b genes of *L. major* and *L. tropica* from Iran and some regions of the world were analyzed and evaluated. According to the results, Cyt b gene was more sensitive than ITS-rDNA. T_m_ for Cyt b gene was lower than ITS-rDNA and could identify the species DNA. The length of ITS-rDNA fragment was 160 bp, meanwhile, that of Cyt b fragment was 866 bp. 

**FIGURE 2:**
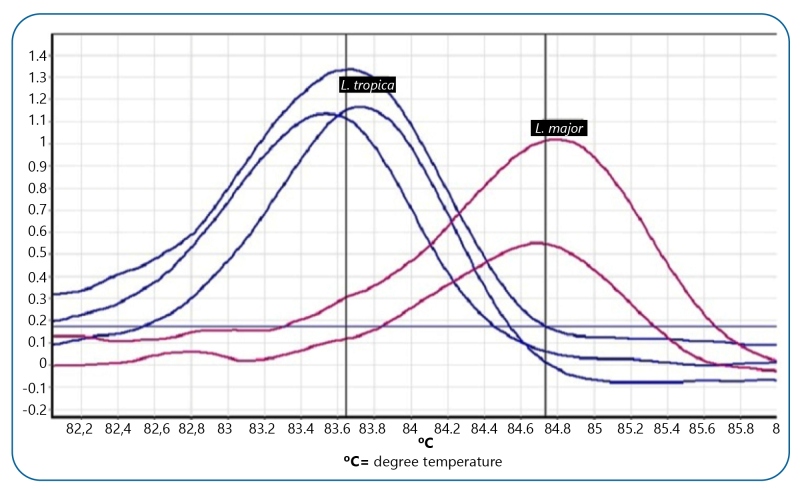
Derivative melt curve analysis of ITS2 gene in different species of isolated *Leishmania* parasites using the HRM assay.

For both *Leishmania* species, amplifying, genotyping, and sequencing of amplified product were performed using newly designed primers. HRM assay managed to detect both species of *Leishmania* and could determine similar *Leishmania* species in the New and Old World. Each melting profile (keeping the same chemical environment with similar reagents and DNA concentrations) was considered for consistency examination. Six consecutive dilutions of DNA template and sequencing dilution reactions were prepared to optimize real-time PCR assay. Duplicates of consecutive dilutions with a DNA-free reaction template (negative control) were prepared to find the specific temperature for separating each *Leishmania* species using Cyt b*.* At the end of the reaction, the standard curve was plotted. Temperature was drawn on a horizontal axis and CT on a vertical axis. After proliferation of DNA dilutions, *L. major* and *L. tropica* were traced to the standard curve (Figure 2, Table 2). 

**TABLE 2:** Serial dilution of plasmid copy numbers (10^6^-10^1^) of two *Leishmania* parasites using Cyt b to find specific temperature for separating of each *Leishmania* species.

Serial dilution of No. of copies	HRM (CT)	
	*L. tropica*	*L. major*
	K27	L.m
**10** ^6^	20.4	-ve
10^5^	23.5	-ve
10^4^	25.2	12
10^3^	28.5	16
10^2^	31.85	18.48
10^1^	35.3	22.46

*Not validated: CT is more than what is acceptable

**FIGURE 3:**
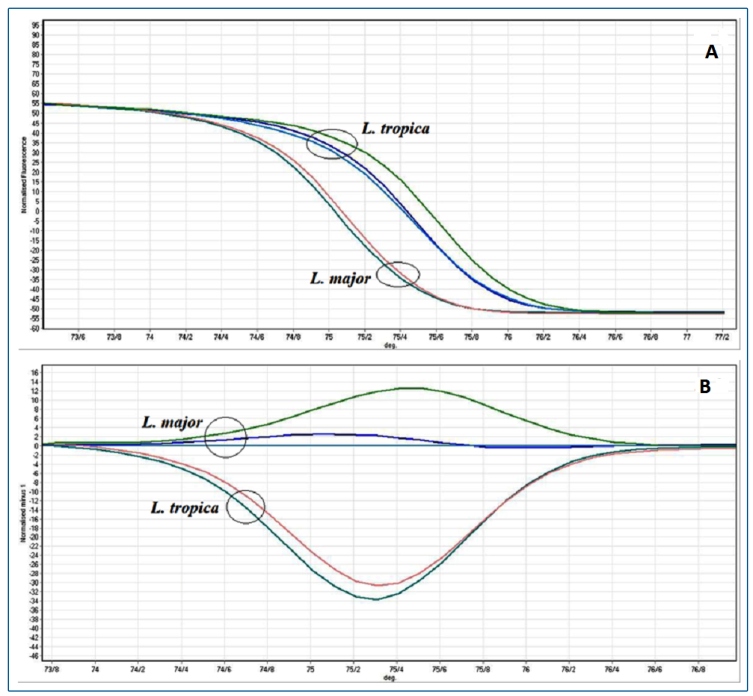
Normalized melting profiles obtained with the HRM assays for *Leishmania major* and *Leishmania tropica*. Each species was tested in triplicate. **A.1:** normalized melting curves for both species; **A.2:** a difference plot for both species.

The melting characteristics of amplicons were assessed for *Leishmania* species by plotting two different curves (Figure 3A,B). In this research, aligned melt curve, the normalized fluorescence curve, and difference plot melt curve were produced to easily distinguish each *Leishmania* species. T_m_ for *L. major* and *L. tropica* was 84.7 °C and 83.6 °C, respectively, which were separated by less than 0.9 °C of temperature difference. 

### The sensitivity of HRM based on serial dilution and drawing standard curve

Different dilutions of DNA template were prepared for validation, accuracy, and sensitivity of HRM assay. High sensitivity of HRM could withdraw minimum amounts of DNA to draw appropriate melting curve. Serial dilutions of *Leishmania* were experienced on three consecutive days with similar reactions. Identical DNA concentrations were repeated with the Cyt b gene. The melting curves for serial dilutions were compared. Identical DNAs were analyzed and melt patterns were appropriated for both species in different days (**Figure 2**).

## CONCLUSIONS

Truthful, quick, and delicate diagnostic procedures are essential for detecting and characterizing causative CL for accurate treatment, precise prognosis, and understanding epidemiology of various species of *Leishmania* parasites^15^ ^,^ ^21^ ^,^ ^22^. 

Phylogenetic and evolutionary analysis should investigate different clinical lesions for avoiding misdiagnosis of leishmaniasis, and helping investigators with the development of molecular epidemiological investigations that can be appropriate for extirpating *Leishmania* parasites in diverse regions^23^ ^,^ ^24^. 

Thus, designing of primers for distinguishing causative agents of leishmaniasis is necessary to find an application with high sensitivity, efficiency, and accuracy^2^ ^,^ ^5^ ^,^ ^19^. In healthcare and research centers of endemic foci of the disease, which have a high number of patients and patrons, accuracy and speed are of great importance^7^ ^,^ ^11^ ^,^ ^24^. Isoenzyme analyses was a gold standard method for typifying *Leishmania* species, but recently, PCR, RFLP, sequencing, and phylogenetic analysis, and now, HRM method using qPCR are more acceptable. No distinct extensively accepted method is available to identify *Leishmania* species and strains today. A valuable molecular technique is needed to be developed to separate *Leishmania* parasites in primary infections^1^ ^,^ ^4^ ^,^ ^7^.

Real-time PCR (qPCR) is convenient, fast, simple, and cost-effective and is widely used in researches for accurate identification and quantification of pathogens. qPCR with high accuracy, sensitivity, specificity, reliability, and availability has been employed for gene scanning, genotyping, and recognition of dissimilar species of *Leishmania* ^25^ ^,^ ^26^. HRM technique has been recorded in recent years to recognize and classify parasitic diseases including leishmaniasis^21^ ^,^ ^23^ ^,^ ^27^. HRM can determine unique curves and detect single nucleotide polymorphisms according to a few differences in the nucleotide content of *Leishmania* parasites^28^. 

Also, previously New World *Leishmania* have been identified and analyzed by qPCR and HRM^29^. HRM is a new approach to real-time PCR; working with HRM requires experience and skills to interpret the results^3^. The third generation EvaGreen is a double-stranded DNA-attached color, and one of its characteristics is the ability to inhibit PCR in comparison with SYBR green I. EvaGreen is also suitable for HRM^30^ ^,^ ^31^.

HRM was employed to identify the *L. major, L. tropica, L. donovani*, and *L. infantum* species by targeting kDNA minicircle, ITS-rRNA, SSU rDNA, g6pd, and hsp70 genes; these have been identified in Eurasia and South America^3^ ^,^ ^24^. By targeting parasite ITS-rDNA and Cyt b genes, the most famous *Leishmania* species were confidently identified using modified and developed HRM as an efficient and cost-effective method^2^ ^,^ ^8^.

In this research, Cyt b and ITS-rDNA genes were targeted, and HRM technique was modified to be more trustworthy, strong, slight, and obvious, to allow the correct genotyping of *Leishmania* species^22^ ^,^ ^32^. In this method, new design of dedicated primers has been one of the most important parts of the work. New designed primers (more economical matter and efficiency with high sensitivity in patients) were considered as advantages of this method. Melting curves provide different temperatures with new primers designed to isolate *Leishmania* species and strains using the ITS-rDNA gene with short repeat (STR) microsatellite employing the HRM method. The prediction results were corrected using CLC software. The altered regions could establish a desirable temperature difference in species and strains of *Leishmania* parasites^19^ ^,^ ^27^. 

Both genes were sequenced to recognize *L. major* and *L. tropica* and had clear sequences of readings. If a mutation occurs in the housekeeping genes (ITS-rDNA), it leads to create a new species of *Leishmania*. These genes are in charge of conserving primary cellular function and their expression is crucial for cell survival^33^. The sequences of our fragment were 160 bp in the ITS-rDNA gene, which were analyzed and evaluated against control samples. 

To validate ITS-rDNA gene, the results were compared with sequences of NCBI database showing *L. major* (accession number: HQ591339) with a similarity of 96% and *L. tropica* (accession number: KT 972151.1) with a similarity of 86.59%. The sensitivity and specificity of primers were designed by BLAST program whereby ITS-rDNA sequences revealed similarity of about 100% with both *L. major* and *L. tropica*. Melting curve analysis was also used to distinguish these parasites^22^ ^,^ ^32^. In expression Cyt b, evolution occurred while no evolution was observed in ITS-rDNA.

Accordingly, we can conclude that HRM assay has many advantages over other genotype methods, including low costs as well as homogeniety, accuracy, and fast speed in a closed tube. Due to its ease of use and rapidness, it is suitable for detection as a quick and low-cost method. HRM is a relatively new technique that provides a clear feature of PCR amplicons. In this method, the changes in fluorescence light that are emitted to DNA at conversion time of a two-stranded DNA into a single-stranded DNA, are measured and can be distinguished from single-nucleotide polymorphism (SNP)^31^.

Our experimental results revealed that the HRM method using EvaGreen and applying ITS-rDNA and Cyt b genes is not only definite and slight, but also repeatable and accurate, and can be intended as a technique for the early recognition and approval of a *Leishmania* infection.
